# Acquired resistance to BRAF inhibition induces epithelial-to-mesenchymal transition in BRAF (V600E) mutant thyroid cancer by c-Met-mediated AKT activation

**DOI:** 10.18632/oncotarget.13480

**Published:** 2016-11-21

**Authors:** Hyung Kwon Byeon, Hwi Jung Na, Yeon Ju Yang, Sooah Ko, Sun Och Yoon, Minhee Ku, Jaemoon Yang, Jae Wook Kim, Myung Jin Ban, Ji-Hoon Kim, Da Hee Kim, Jung Min Kim, Eun Chang Choi, Chang-Hoon Kim, Joo-Heon Yoon, Yoon Woo Koh

**Affiliations:** ^1^ Department of Otorhinolaryngology, Yonsei University College of Medicine, Seoul, Republic of Korea; ^2^ Department of Pathology, Yonsei University College of Medicine, Seoul, Republic of Korea; ^3^ Department of Radiology, Yonsei University College of Medicine, Seoul, Republic of Korea; ^4^ Brain Korea 21 Plus Project for Medical Science, Yonsei University College of Medicine, Seoul, Republic of Korea; ^5^ YUHS-KRIBB Medical Convergence Research Institute, Seoul, Republic of Korea; ^6^ Department of Otorhinolaryngology, Soonchunhyang University College of Medicine, Republic of Korea; ^7^ Department of Otorhinolaryngology-Head and Neck Surgery, Yonsei University Wonju College of Medicine, Wonju, Republic of Korea; ^8^ The Airway Mucus Institute, Yonsei University College of Medicine, Seoul, Republic of Korea; ^9^ Research Center for Human Natural Defense System, Yonsei University College of Medicine, Seoul, Republic of Korea

**Keywords:** thyroid cancer, molecular targeted therapy, drug resistance, BRAF mutation, epithelial-mesenchymal transition

## Abstract

Previously, the authors have identified that c-Met mediates reactivation of the PI3K/AKT pathway following BRAF inhibitor treatment in BRAF (V600E) mutant anaplastic thyroid cancer, thereby contributing to the acquired drug resistance. Therefore dual inhibition of BRAF and c-Met led to sustained treatment response, thereby maximizing the specific anti-tumor effect of targeted therapy. The present study goes one step further and aims to investigate the effect of acquired resistance of BRAF inhibitor on epithelial-to-mesenchymal transition (EMT) in BRAF mutant thyroid cancer cells and the effect of dual inhibition from combinatorial therapy. Two thyroid cancer cell lines, 8505C and BCPAP were selected and treated with BRAF inhibitor, PLX4032 and its effect on EMT were examined and compared. Further investigation was carried out in orthotopic xenograft mouse models. Unlike BCPAP cells, the BRAF inhibitor resistant 8505C cells showed increased expressions of EMT related markers such as vimentin, β-catenin, and CD44. The combinatorial treatment of PLX4032 and PHA665752, a c-Met inhibitor reversed EMT. Similar results were confirmed *in vivo*. c-Met-mediated reactivation of the PI3K/AKT pathway contributes to the drug resistance to PLX4032 in BRAF (V600E) mutant anaplastic thyroid cancer cells and further promotes tumor cell migration and invasion by upregulated EMT mechanism. Dual inhibition of BRAF and c-Met leads to reversal of EMT, suggesting a maximal therapeutic response.

## INTRODUCTION

Thyroid cancer is the most common endocrinologic malignancy. Multiple genetic mutations or alterations are involved in the tumorigenesis of thyroid cancer, BRAF (V600E) mutation being the most common [1, 2]. BRAF (V600E) mutation causes sustained BRAF kinase activity leading to constitutive activation of MAPK cascade pathway, and BRAF (V600E) mutation bearing thyroid cancer presents aggressive clinicopathologic features. BRAF (V600E) mutation is also commonly detected in other tumors such as melanoma and colorectal cancer [3, 4]. In malignant melanoma, a FDA approved selective BRAF inhibitor vemurafenib (PLX4032) has shown high treatment response [5]. However, unlike in metastatic melanoma, the treatment effects of vemurafenib in BRAF (V600E) mutant thyroid cancer are often insignificant.

Previously, the authors have elucidated to a considerable level the causative mechanism underlying acquired resistance to BRAF inhibition in BRAF (V600E) mutant thyroid cancer [6]. The previous study demonstrated differential treatment responses of BRAF inhibition in different types of thyroid cancer harboring BRAF (V600E) mutation. On the contrary to the favorable treatment response in BCPAP, a papillary thyroid cancer (PTC) cell line, decreased treatment response was noted in 8505C, an anaplastic thyroid cancer (ATC) cell line. It has been identified that c-Met mediates reactivation of the PI3K/AKT pathway thereby contributing to the acquired resistance of BRAF inhibition in BRAF mutant ATC cells. Therefore dual inhibition of BRAF and c-Met led to sustained treatment response, thereby maximizing the specific anti-tumor effect of targeted therapy.

Epithelial-to-mesenchymal transition (EMT) is a process where epithelial cells demonstrate changes in their morphology and motile behavior as they differentiate into mesenchymal cells. During the EMT, epithelial cells lose cell to cell contact, undergo cytoskeleton remodeling, acquire expression of mesenchymal components, and manifest a migratory phenotype [7, 8]. It is generally accepted that EMT plays a crucial role in the migration, invasion, and metastasis of cancer.

On the basis of our previous findings, this present study goes one step further and aims to investigate the effect of acquired resistance of BRAF inhibitor on EMT in BRAF mutant thyroid cancer cells. Also we investigated the effect of dual inhibition from combinatorial therapy on EMT in BRAF inhibitor-resistant ATC.

## RESULTS

### Differential effect against EMT is shown in different BRAF (V600E) mutant thyroid cancer cell lines to PLX4032 treatment

**Figure 1:**
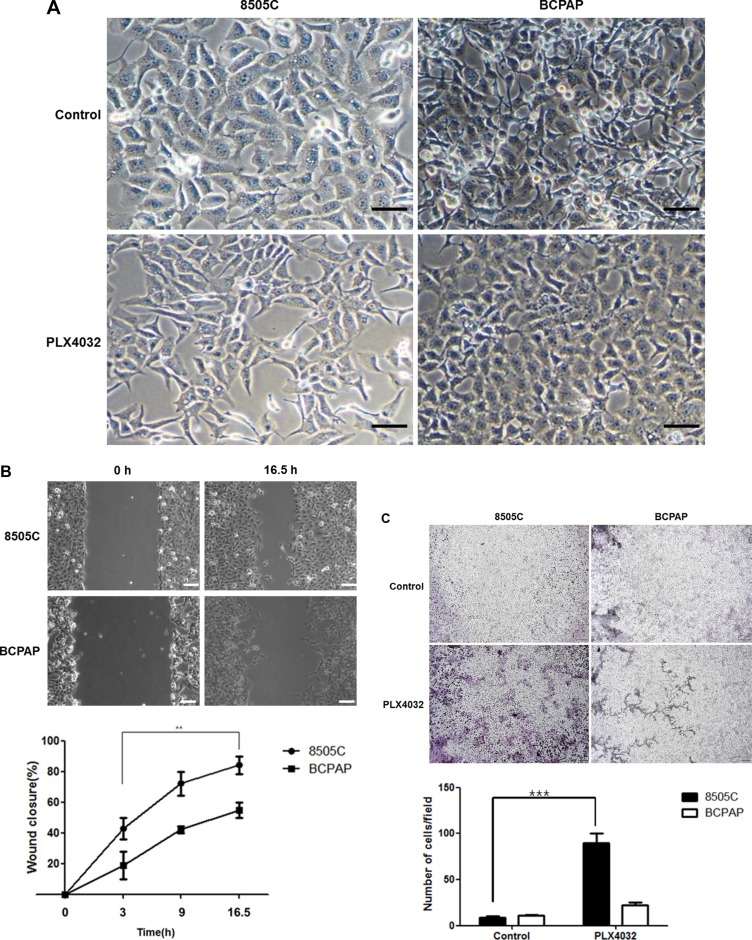
Differential effect against EMT in BRAF (V600E) mutant thyroid cancer cell lines 8505C and BCPAP to PLX4032 treatment (**A**) Observation under light microscopy (x20) after treatment with 1 μM PLX4032 for 9 h. (**B**) Live wound healing assay after treatment with 1 μM PLX4032 for 9 h. **p < 0.01. (**C**) Analysis of cell invasion with transwell migration assay after treatment with 1 μM PLX4032 for 9 h. ***p < 0.001.

To evaluate the differential effects of EMT in two different BRAF (V600E) mutant thyroid cancers, PLX4032 was treated to 8505C and BCPAP cells which have each been previously verified as BRAF inhibitor-resistant and -sensitive cell lines, respectively. Simple examination under light microscopy (magnification x20) after PLX4032 treatment revealed distinct morphological differences between 8505C and BCPAP cells. That is, the 8505C cells presented long, spindle shaped phenotypes resembling mesenchymal cell morphology whereas the BCPAP cells were more closely aggregated together with more round shapes suggesting epithelial cell phenotype (Figure 1A). From the live wound healing assays of 8505C and BCPAP cell lines after PLX4032 treatment, the rate of wound closure was prominently faster in 8505C cells (Figure 1B, Supplementary Video S1). This increased EMT trend could also be verified in transwell migration assay, where cell invasion was significantly increased in 8505C cells compared to BCPAP cells (Figure 1C).

**Figure 2:**
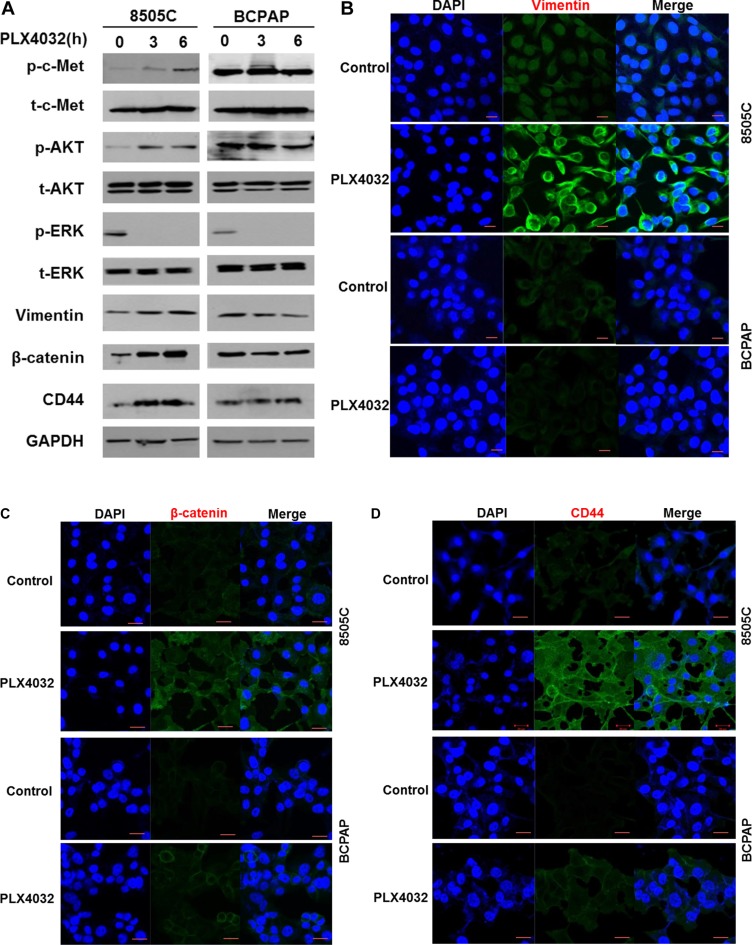
Expression of EMT related markers in 8505C and BCPAP to 1 μM PLX4032 treatment for 9 h (**A**) Western blot analysis after PLX4032 treatment in 8505C cells. (**B**) Immunofluorescence confocal microscopy of vimentin. (**C**) Immunofluorescence confocal microscopy of β-catenin. (**D**) Immunofluorescence confocal microscopy of CD44.

Western blot analysis in 8505C and BCPAP cells following PLX4032 treatment revealed that p-c-Met and p-AKT levels were significantly increased in 8505C cells together with increased levels of vimentin, β-catenin, and CD44. These markers however, were unchanged in BCPAP cells (Figure 2A). Increases of EMT related markers in 8505C were also confirmed in immunofluorescence confocal microscopy where vimentin, β-catenin, and CD44 expressions were all increased in 8505C cells after PLX4032 treatment whereas there was no change in BCPAP cells (Figure 2B–2D).

### PLX4032 treatment increases EMT via over-expression of PI3K/AKT pathway mediated by p-c-Met in 8505C

**Figure 3:**
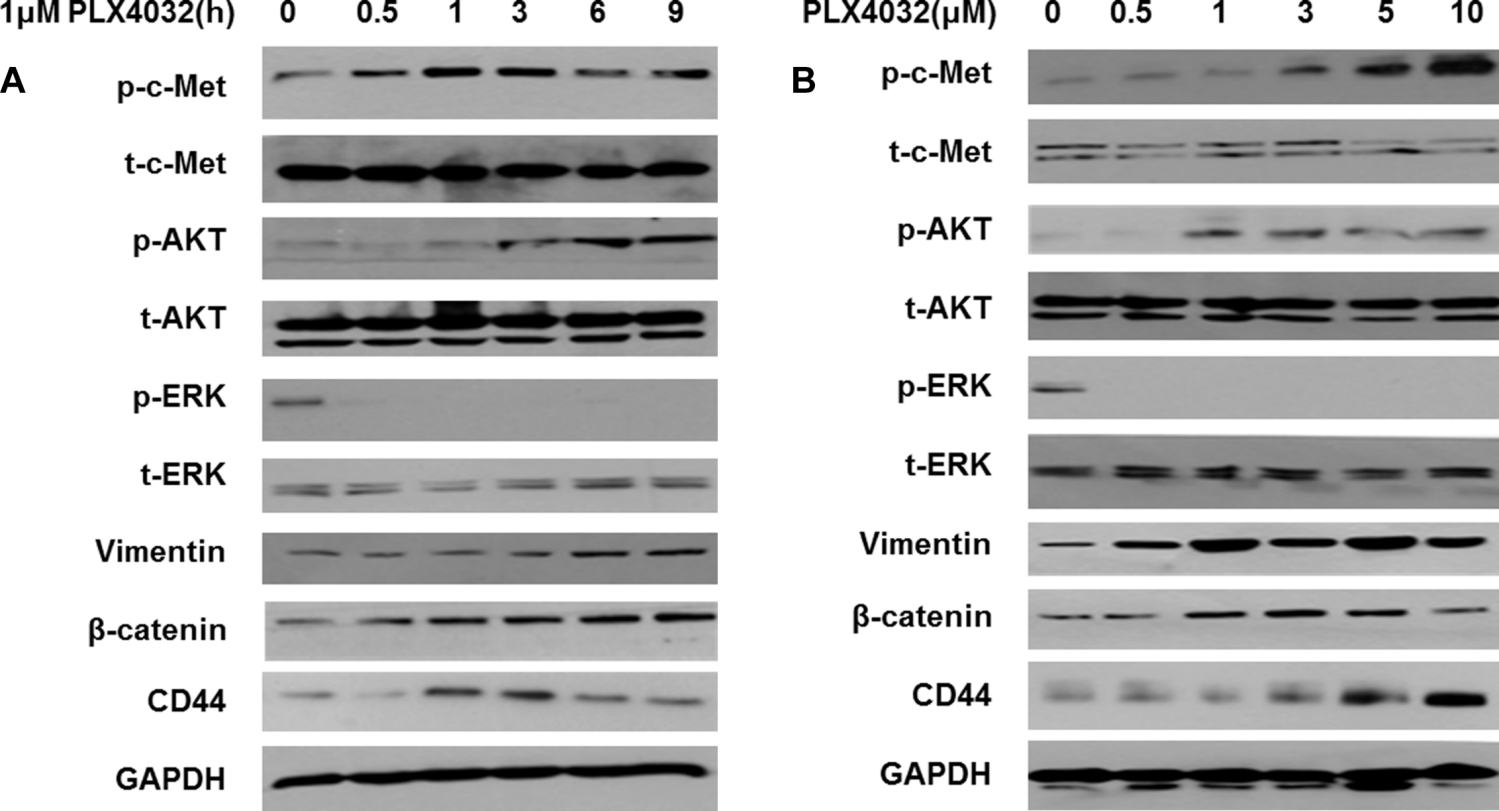
PLX4032 treatment increases EMT via over-expression of PI3K/AKT pathway mediated by p-c-Met in 8505C (**A**) Western blot analysis after treatment of 1 μM PLX4032 of increasing treatment times in 8505C cells. (**B**) Western blot analysis after treatment of PLX4032 of increasing dosages for 6 h in 8505C cells.

In order to investigate the EMT changes of 8505C cells under BRAF inhibition, PLX4032 was treated to 8505C cells at different times and different concentrations. According to increased treatment times of PLX4032, p-c-Met expression was significantly increased followed by increased levels of p-AKT (Figure 3A). Also, markers of EMT such as vimentin, β-catenin, and CD44 were consequently increased. The p-c-Met mediated PI3K/AKT pathway activation leading to over-expression of EMT markers were also confirmed after treatment of PLX4032 in a dose-dependent manner (Figure 3B).

### Dual inhibition of BRAF and c-Met has reversal effect on EMT in 8505C

**Figure 4:**
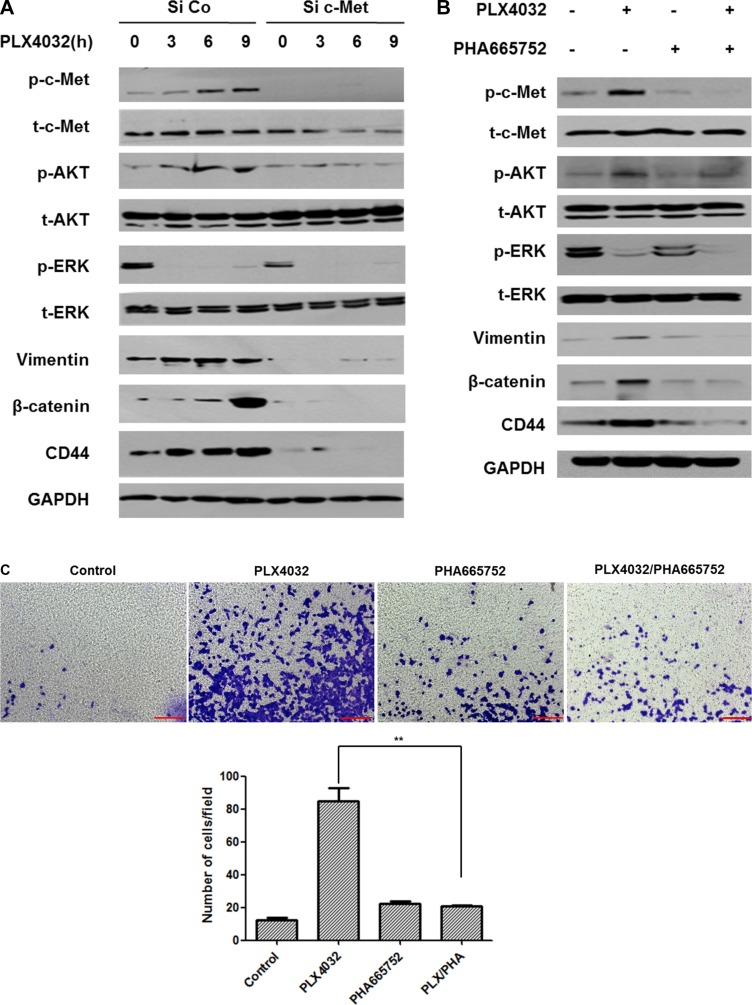

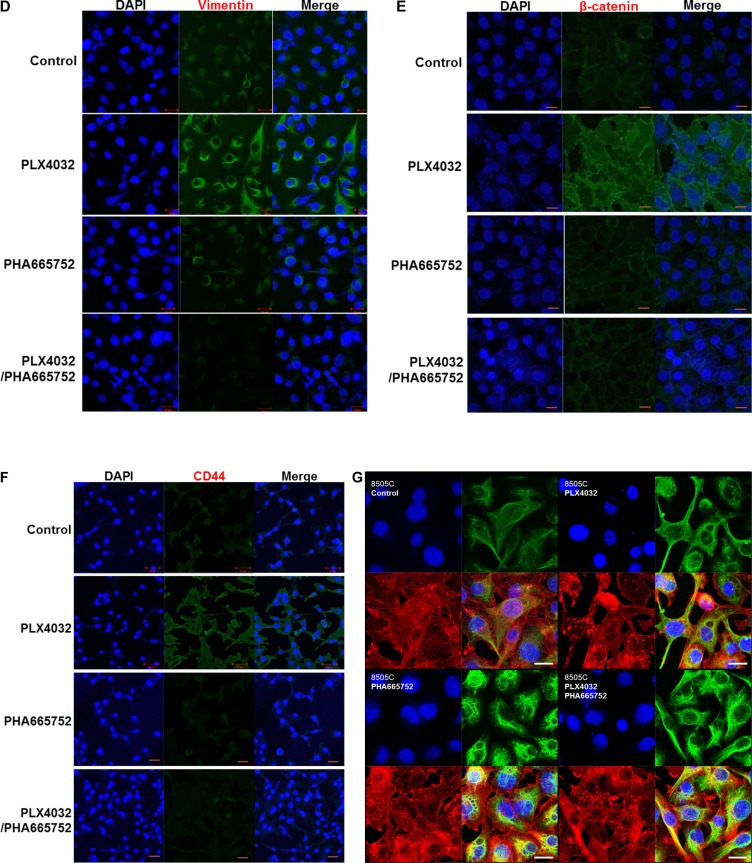
Dual inhibition of BRAF and c-Met has reversal effect on EMT in 8505C cells (1 μM PLX4032, 0.5 μM PHA665752) (**A**) 8505C cells transfected with small interfering RNA (siRNA) of c-Met or negative control siRNA were treated with 1 μM PLX4032 for 3,6, and 9 h. (**B**) Western blot analysis after different drug treatment conditions for 9 h. (**C**) Transwell migration assay of 8505C cells under each different treatment conditions. **p < 0.01. (**D**) Immunofluorescence confocal microscopic examination of vimentin expression under different drug treatment conditions. (**E**) Immunofluorescence confocal microscopic examination of β-catenin expression under different drug treatment conditions. (**F**) Immunofluorescence confocal microscopic examination of CD44 expression under different drug treatment conditions. (**G**) 3D confocal microscopic examination of intracellular vimentin network under different drug treatment conditions (Blue, nucleus; red, f-actin; green, vimentin).

When c-Met was knocked down and PLX4032 treated with increasing times, all vimentin, β-catenin, and CD44 expression levels were markedly decreased, together with low expressions of p-c-Met, p-AKT, and p-ERK (Figure 4A).

In accordance with the previous results, vimentin, β-catenin, and CD44 were over-expressed together with increased levels of p-c-Met and p-AKT, following PLX4302 treatment. Whereas there was no change except for the decrease of p-c-Met upon PHA665752 treatment, all expression levels of p-c-Met, p-AKT, p-ERK, and EMT related markers were decreased following combinatorial treatment of PLX4032 with PHA665752 (Figure 4B).

From the transwell migration assay (Figure 4C), cell invasion was prominent in PLX4032 single treatment condition but was not increased following combinatorial treatment of PLX4032 and PHA665752. Under immunofluorescence confocal microscopic examination, vimentin, β-catenin, and CD44 expressions were all increased following PLX4032 single treatment however, all markers were barely detectable following combinatorial treatment of PLX4032 and PHA665752 (Figure 4D–4F). Furthermore, changes in the intracellular network of vimentin according to each drug treatment condition were investigated under 3D confocal microscopy (Figure 4G). That is, the vimentin expression in PLX4032 single treatment condition revealed extensively distributed vimentin network, whereas the intracellular vimentin was reorganized into perinuclear aggregates following PLX4032 and PHA665752 combinatorial treatment.

### Effects on EMT following PLX4032 and PHA665752 treatment in a xenograft mouse model orthotopically injected with 8505C

**Figure 5:**
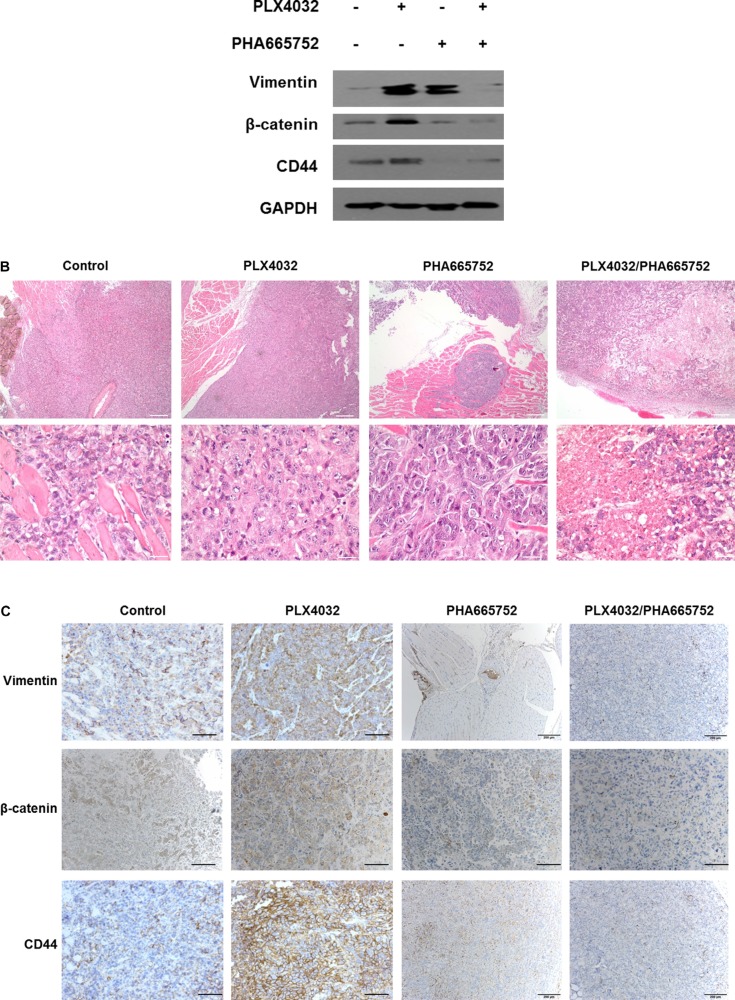
Effects on EMT following PLX4032 and PHA665752 treatment in a xenograft mouse model orthotopically injected with 8505C cells Orthotopic injection of 8505C cells in thyroid glands of BALB/c nude mice were done and randomly divided into four groups with different treatment conditions as follows; DMSO (20 mg/kg/day), PLX4032 (20 mg/kg/day), PHA665752 (10 mg/kg/day), and combinatorial PLX4032 (20 mg/kg/day) and PHA665752 (10 mg/kg/day) treatment. (**A**) Western blot analysis of intratumoral protein expression from the obtained tumor specimens in each group. (**B**) Histopathological analysis by H & E staining from tumor tissue samples obtained from mice of each group. (**C**) Immunohistochemistry of tumor tissues from each treatment group.

The tendencies shown in *in vitro* experiments were also confirmed in *in vivo* animal studies. In concordance with our previous study results, the tumor volumes and weights were paradoxically larger in the mice of PLX4032 single treatment group compared to the control group, but both values were significantly smaller in the combinatorial treatment group (data not shown). Considering these changes in tumor size for each treatment groups, EMT protein expression levels in tumor specimens of each group were analyzed. All vimentin, β-catenin, and CD44 expressions were increased in PLX4032 single treatment group but the levels of EMT markers were considerably decreased in combinatorial drug treatment group (Figure 5A).

Histopathological analysis by hematoxylin and eosin (H & E) staining from tumor specimens of each treatment group revealed aggressive pathologic features in PLX4032 single treatment group and favorable pathologic features in combinatorial drug treatment group, with reference to control (Figure 5B). More specifically, the PLX4032 single treatment group showed large sized tumor mass with local infiltration but the combinatorial treatment group showed small sized tumor mass with extensive necrosis and partial formation of fibrotic tumor capsule under low power examination. The control group presented medium sized mass showing infiltration into adjacent skeletal muscle with only focal necrosis. Under high power microscopic examination, viable anaplastic tumor cells were noted in control and PLX4032 single treatment groups however, the combinatorial treatment group presented cellular degenerative changes and decreased cell viability with extensive necrosis.

Immunohistochemistry demonstrating intratumoral EMT-related proteins expression levels of each treatment groups confirmed high detection levels of all vimentin, β-catenin, and CD44 in PLX4032 single treatment group compared to control (Figure 5C). However the expressions of all EMT markers were markedly decreased in combinatorial drug treatment group.

## DISCUSSION

ATC accounts less than 5% of all thyroid carcinomas and is considered as a highly virulent malignancy with an extremely poor prognosis [9, 10]. It presents an overall median survival time of 6 months despite the best multidisciplinary care and it is associated with an even graver prognosis in metastatic disease [11]. Even the differentiated thyroid carcinomas which generally have an excellent prognosis, show disappointing treatment outcomes in patients with extensive local invasion or distant metastases [12, 13]. The 5-year survival rate is merely 50% in patients who presents distant metastases at initial diagnosis [14, 15]. The EMT mechanism is considered important in the initiation and promotion of cancer invasion and metastasis. The hallmarks of EMT include downregulation of epithelial markers and upregulation of mesenchymal markers, together with morphological and functional changes of the cell. So as the epithelial cells lose their apical polarity and cell-cell contact and acquire spindle-shaped, fibroblastic cell phenotypes with increased cell motility, expressions of N-cadherin, vimentin, fibronectin, and osteopontin are increased while expression of E-cadherin is decreased [16–20]. Also, transcription factors including Snail, Slug, and Twist are activated with nuclear localization of β-catenin and increased expression of CD44, a stemness marker implicated in migration and metastasis of cancer cells [21].

Multiple complex mechanisms are involved in the EMT process and evidence suggests that the PI3K/AKT signaling pathway plays a mechanistically important role in migration and progression of tumors including thyroid cancer [22, 23]. In particular, AKT has shown to activate cell migration and invasion in thyroid cancer cell lines [20, 24]. That is, intranuclear accumulation of p-AKT and nuclear exclusion of p27 to the cytoplasm was directly associated with the invasiveness of thyroid cancer cells with acquisition of mesenchymal phenotype, thereby suggesting AKT activation is involved in direct induction of EMT. Furthermore, this has been suggested by many others where levels of Snail and Twist and AKT phosphorylation were also positively correlated in oral squamous cell carcinoma and prostate carcinoma [25–27]. In head and neck squamous cell carcinoma, PI3K/AKT activation degrades E-cadherin and promotes cell invasion and migration [28, 29]. In gastric cancer and breast cancer cells, inhibition of PI3K/AKT signaling pathway repressed MMP2/MMP9 activation and reduced EMT [30, 31]. The PI3K/AKT pathway positively regulates the Wnt/β-catenin signaling pathway by the increase of intracellular β-catenin levels from AKT phosphorylation which then induces the movement of β-catenin into the nucleus to bind with TCF/LEF. This binding ultimately leads to activation of genes targeted to induce EMT [29, 32]. Also, there have been earlier reports that hepatocyte growth factor (HGF) and its receptor c-Met are overexpressed in thyroid cancer and HGF treatment increases thyroid cancer cell motility through overexpression of EMT markers [33–36]. The PI3K/AKT pathway is also closely related, since it is indispensable for HGF-induced EMT [37, 38].

In the previous study, the c-Met mediated reactivation of PI3K/AKT pathway following PLX4032 treatment was identified as the key mechanism underlying the resistance to BRAF inhibition in ATC [6]. In the present study, it could be confirmed that the acquired resistance mechanism also induced the EMT process. That is, unlike the BRAF inhibitor-sensitive BCPAP cells, the BRAF inhibitor-resistant 8505C cells presented upregulated EMT following PLX4032 treatment. The fundamental causative mechanism for this differential drug response in different types of BRAF (V600E) mutant thyroid cancer is unknown and is beyond the scope of this study but further investigation is warranted. Both morphological and functional features of mesenchymal phenotypes were verified, together with elevated expression of EMT related markers such as vimentin, β-catenin, and CD44 (Figures 1, 2). Furthermore, the upregulation of EMT markers following PLX4032 treatment in 8505C were in accordance to increased levels of p-c-Met and p-AKT (Figure 3) and conversely, expressions of these EMT related markers were uniformly decreased both by c-Met knockout and c-Met inhibitor treatment together with BRAF inhibition (Figure 4). The EMT reversal effect following combinatorial treatment of PLX4032 and PHA665752 could also be confirmed by the decreased cell invasion ability (Figure 4). Besides the expressional changes, the intracellular network reorganization of vimentin from EMT is also considered important [39]. In accordance to previous reports, further investigation of the vimentin network revealed generalized intracellular distribution of vimentin in upregulated EMT state however, the intracellular vimentin was presented as juxtanuclear caps in EMT reversal status (Figure 4G). Upregulation of EMT caused by the predilection for PI3K/AKT pathway following BRAF inhibition is plausible, considering the aforementioned studies reporting close associations between HGF/c-Met, PI3K/AKT signaling and EMT. Already preliminary results of combinatorial treatment of BRAF inhibitor with an AKT inhibitor in this setting confirming similar therapeutic responses have been obtained (data not shown). The results were also verified *in vivo* (Figure 5).

There have been previous reports that BRAF (V600E) mutation stimulates migration and invasion of thyroid cancer cells, through the increased expression of EMT-related markers via a MEK/ERK-dependent mechanism and that these processes together with tumor proliferation can be inhibited by BRAF inhibitor PLX4720 treatment [40, 41]. This however, may not always be the case, as has been demonstrated in this present study. This discrepancy in contrary results may be due to the fact that we have used a different, more clinically available BRAF inhibitor drug, PLX4032 (vemurafenib) and at suboptimal dosage, but nevertheless should receive more attention since the BRAF inhibitor is not always sensitive in BRAF (V600E) mutant thyroid cancer in reality. Various underlying resistance mechanisms against BRAF inhibitor therapy in thyroid cancer have been investigated in previous studies [6, 42]. That is, our findings in the present study would be more applicable to the concept of drug resistance against BRAF inhibitor and would pose more valuable clinical implications. The interesting fact that BRAF inhibition in 8505C cells induces EMT from our study suggests that sustained BRAF inhibitor treatment in thyroid cancer would do more harm than good, which raises an important clinical issue.

Recently it has been shown in numerous reports that aberrant activation of EMT and an associated cancer stem cell phenotype are considered a major cause of drug therapy resistance in aggressive solid tumors such as pancreatic cancer, lung cancer, and melanoma, particularly emphasizing the important role of the EMT-activator, ZEB1 in conferring stemness and resistance [43–46]. This EMT-related phenotypic change and tumor cell plasticity shown in the present study therefore, can be suggested furthermore as another acquired resistance mechanism to BRAF inhibitor, so further in-depth studies are warranted to elucidate the precise underlying mechanism.

In summary, this study demonstrates that the previously confirmed BRAF inhibitor-resistant 8505C cells showed increased EMT response via acquired resistance mechanism mediated by the increased expression of c-Met. Accordingly, the combinatorial treatment of BRAF inhibitor and c-Met inhibitor reversed EMT. To our knowledge, this is the first report showing that acquired drug resistance to BRAF inhibition promotes not only tumor progression and proliferation, but also migration and invasion of BRAF (V600E) mutant thyroid cancer cells through upregulated EMT induced by c-Met-mediated AKT activation. Therefore combinatorial multi-targeted therapy could overcome the limitation of single agent therapy and maximize anti-tumor effect. Furthermore it is expected that the PI3K/AKT signaling pathway will have greater clinical implications by attracting widespread attention as a potential target for the prevention and treatment of metastatic cancer.

## MATERIALS AND METHODS

### Cell culture

BRAF (V600E) mutant thyroid cancer cell lines, 8505C and BCPAP were obtained from DSMZ (German collection of microorganisms and cell cultures, Braunschweig, Germany) and were maintained in 10% RPMI medium (Lonza, Wakersville, USA) and cultured in a humidified incubator at 37°C in an atmosphere containing 5% CO_2_.

### Time-lapse microscopy for wound healing assay

The *in-situ* wound healing potentials of thyroid cancer cells were monitored by live cell imaging microscopy (DMI6000B, Leica, Bensheim, Germany). 8505C and BCPAP cells (1 × 10^5^ cells/well) were seeded in a 6-well plate and incubated for 24 h. When seeded cells were stable in the plate, 8505C and BCPAP cells were each treated with 1 μM PLX4032, and wound was carefully made across the cell monolayer by a plastic pipette tip. The medium was refreshed with complete medium. 1 μg/ml mitomycin C was treated to the cells to prevent the cell proliferation effect. Cells were then incubated in a live cell chamber (Chamlid HX, LCI, Seoul, Republic of Korea), connected to a temperature and CO_2_ controllers (Heating & Cooling system/CU-301 and Gas mixer/FC-5N, LCI, Seoul, Republic of Korea). The migratory behavior of cells was monitored for 2 days after scratching and time-lapse sequential microscopic images were acquired at intervals of 30 min. After the complete acquisition of time-lapse live cell microscopic images, movies were made at 3 frames/sec (scale bars represent 100 μm). Migration rates of 8505C and BCPAP cells were determined by measuring the wound closure percentage as defined below:

Wound closure (%) = (migrated cell surface area/total surface area) x 100, migrated cell surface area = length of cell migration (mm) x 2 x length, total surface area = 2.4 mm x length.

For each time frame, the experiments were repeated three times.

### Transwell migration assay

Cell invasion was determined by transwell migration assay. First, 8505C and BCPAP cells (1 ×10^5^ cells/well) were seeded in a 6-well plate and drug was treated for 9 h. Meanwhile, 70 μl gel mixture of 1:1 ECM gel and 0.2% RPMI was added in a 24-well transwell chamber (Corning, Kennebunk, USA) and was kept at 4°C. After 9 h drug treatment, the cells in the 6-well plate were seeded (3 × 10^4^ cells/100 μl) in the transwell chamber. The chamber was incubated for 6 h with 5% CO_2_ at 37°C and then non-migrant cells left on the upper section of the filter were removed using a cotton swab. Finally, attached cells at the lower section were stained with crystal violet and counted under a light microscope (magnification x 40).

### Confirmation of EMT marker expression and c-Met inhibitor-induced reversal of EMT by western blot analysis

Thyroid cell lines were washed with phosphate-buffered saline (PBS) and were treated with lysis buffer (10 mM Tris-HCl (pH 7.4), 100 mM NaCl, 1 mM EDTA, 1 mM EGTA, 1 mM NaF, 20 mM Na_4_P_2_O_7_, 2 mM Na_3_VO_4_, 1% Triton X-100, 10% glycerol, 0.1% SDS, 0.5% deoxylcholate) (Invitrogen, Camarillo, USA) supplemented with 1 mM PMSF and protease inhibitors cocktail (Sigma-Aldrich, St. Louis, USA) and then harvested. The protein was centrifuged under 13,200 rpm for 10 min and the supernatant was used for Western blot analysis where the protein amount was quantified with Pierce BCA Protein Assay Kit (Thermo, Rockford, USA). The lysates were electrophoresed using 10% SDS-PAGE to separate proteins and electrotransferred to polyvinylidene fluoride membranes (Millipore, Schwalbach, Germany). An equal amount of protein (30 μg) was loaded per well and the proteins were transferred onto the following antibodies to be left at 4°C overnight incubation: p-c-Met (1:1000), p-AKT (1:1000), p-ERK (1:1000), vimentin (1:1000), β-catenin (1:1000), CD44 (1:1000), and GAPDH (1:1000). The next day, it was thoroughly washed with TBS containing 0.1% Tween-20, and then reacted with secondary rabbit antibody (Jackson, West Grove, USA) and anti-mouse antibody (Jackson) to be visualized on X-ray film using SuperSignal West Pico Chemiluminescent Substrate (Thermo).

### Immunofluorescence confocal microscopy

Cover glasses were coated in a 6-well plate and 1 × 10^5^ cells were seeded. After 24 h, drug was treated to the seeded cells for 9 h. Then the media was removed and the cells were washed twice with PBS. Cells were then fixed in 1% paraformaldehyde (PFA) at room temperature for 15 min and washed twice. Next the cells were blocked with mixture of PBS, 1% bovine serum albumin (BSA), and 10% normal goat serum for 1 h and then treated with the following primary antibodies, followed by overnight incubation: vimentin (1:200), β-catenin (1:200), and CD44 (1:200). The next day, it was thoroughly washed twice with PBS and then each reacted with the following secondary antibodies (1:500) for 2 h: Alexa Flour 488 donkey anti-mouse antibody (Invitrogen) and Alexa Flour 488 donkey anti-rabbit antibody (Life Technology). After further washing with PBS, 10 μl moutin was treated on the cover glasses and transferred to slides. The stained cells were then visualized with confocal microscopy (Carl Zeiss, Oberkochen, Germany) at 488 nm.

### Transient transfection of c-Met and RNA interference (siRNA)

3 × 10^4^ cells per well were seeded onto 6-well culture dishes containing 2 ml antibiotic-free medium supplemented with 10% fetal bovine serum (FBS). Next, 500 μl optimum and 10 μl RNAiMAX, and 500 μl optimum and 5 μl RNAi were mixed and left at room temperature for 5 min, and then mixed together. siRNAs for the control and c-Met (BIONEER sense: 100451 antisense:100451) were then mixed and after 20 min, droplets of the mixture were applied at the cells and left to react for 6 h. Thereafter, the medium was changed to 10% FBS and penicillin-streptomycin supplemented medium and the cells were incubated at 37°C with 5% CO_2_ for 48 h. Subsequently PLX4032 was treated for 9 h and the cells were harvested and confirmed by Western blot analysis.

### 3D Confocal microscopy

8505C cells were plated on coverslips at a cell density of (1 × 10^5^) in a 6-well plate with 18 mm cover slip and allowed to adhere overnight. On the following day, 8505C cells were incubated in serum starved medium for 24 h to induce cell cycle arrest. The cells were then fixed for 30 min with 4% PFA. Subsequently, the cells were washed three times with PBS and permeabilized with 0.5% Triton X-100 for 15 min. The cells were washed three times with blocking buffer (PBS supplemented with 0.1% BSA and 0.001% sodium azide) and incubated for 30 min. The cells were further incubated for 30 min at 22°C with primary antibodies at a 1:200 dilution. After primary serum incubation, the cells were washed three times with blocking buffer and incubated with secondary antibodies at a dilution ratio of 1:500 for 30 min at 22°C. Finally, the cells were washed again for three times with PBS. A nucleus staining solution with Hoechst 33342 (Cat #. H3570, Molecular Probes, Waltham, MA, USA) was added to a final concentration of 5 mg/mL. The confocal microscopic images were obtained using a confocal microscope (LSM-700, Carl Zeiss, Jena, Germany) with a 63× objective and ZEN software (version 5.5.0.375, Carl Zeiss), which was designed for acquisition and processing of confocal microscopic images.

### Orthotopic xenograft mouse model

8505C cells were harvested and 1 × 10^5^ cells were suspended in 5 μl PBS which were then injected orthotopically in the right thyroid gland of male athymic nude BALB/c mice, aged 6 weeks (Orientbio Inc., Seongnam-si, Korea) with a 25 μl Hamilton syringe (Hamilton Company, Reno, NV). Tumor formation was examined at 3 weeks post injection, and the mice were randomly divided into four groups of four. The control group was treated with DMSO and the other 3 groups were each injected with PLX4032, PHA665752, PLX4032 and PHA665752 combination respectively, 3 times a week for 3 weeks. The mice were then euthanized and Western blot analysis, H&E staining, and immunohistochemistry analysis were conducted from specimens. All mice experiments were approved by the Committee for ethics in animal experiments of Yonsei University College of Medicine and all experimental mice were handled in accordance with the Guide for the care and use of laboratory animals in Department of laboratory animal resources, Yonsei University College of Medicine.

### Immunohistochemistry analysis

The tumor tissue specimens obtained from mice were processed for paraffin section and deparaffinized consequently with xylene and ethanol. Antigen retrieval was done for 15 min followed by reaction with H_2_O_2_ for 10 min and blocking (10% normal goat serum + 0.01% BSA + dilution) for 1 h. The slides were then incubated overnight at 4°C with primary anti-vimentin, β-catenin, and CD44 antibody (1:200). Next, the slides were treated with secondary anti-rabbit antibodies (1:500) for 1 h and reacted using DAB histochemistry kit (Life technologies, Rockford, IL). Finally the slides were stained with hematoxylin and processed with mounting solution (DAKO) and visualized using a Nikon light microscope. Microscopic images were captured and processed using AxioCam digital microscope camera and AxioVision Image processing software (Carl Zeiss Vision, Oberkochen, Germany).

### Statistical analysis

All data were obtained from triplicate independent experiments, and the parameters were represented as means ± SD. Student's *t-test* and one-way ANOVA were performed using SPSS 20.0 statistical software (SPSS, Chicago, IL, USA). A *p* < 0.05 was considered statistically significant (*p < 0.05; **p < 0.01; ***p < 0.001).

## SUPPLEMENTARY MATERIALS


