# Supplementary material for: Acquired resistance to BRAF inhibition induces epithelial-to-mesenchymal transition in BRAF (V600E) mutant thyroid cancer by c-Met-mediated AKT activation
Source: Oncotarget. 2016 Nov 21;8(1):596–609. doi: 10.18632/oncotarget.13480 (PMC5352181; doi:10.18632/oncotarget.13480)
Supplement: Supplementary file 1 [file oncotarget-08-596-s001.pdf]

Acquired resistance to BRAF inhibition induces epithelial-to-mesenchymal transition in BRAF (V600E) mutant thyroid cancer by c-Met-mediated AKT activation

Supplementary Materials

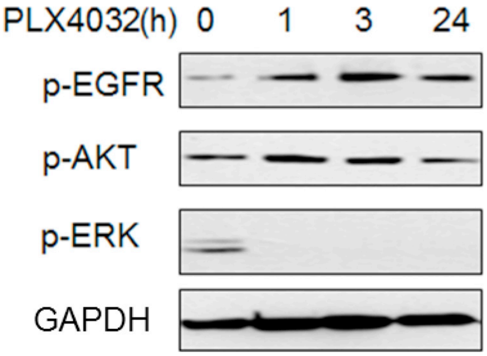

Supplementary Figure S1: Western blot analysis after treatment of 1 μM PLX4032 until 24 hours in 8505C cells. p-EGFR expression was increased following BRAF inhibition.

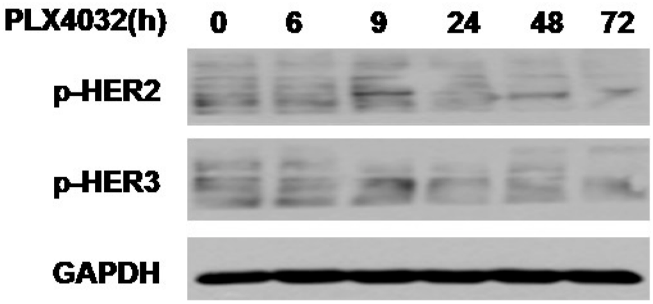

Supplementary Figure S2: Western blot analysis after treatment of 1 μM PLX4032 until 72 hours in 8505C cells. There were no significant changes in p-HER2 or p-HER3 following BRAF inhibition.

Supplementary Table S1: Microarray data set of 8505C and BCPAP cells

Gene	Fold change
MET	1.6
PIK3CA	–1.2
BRAF	–1.1
CRAF	1.1
ERK	–1.3

The expression of each gene is expressed in fold change values of 8505C with reference to BCPAP.

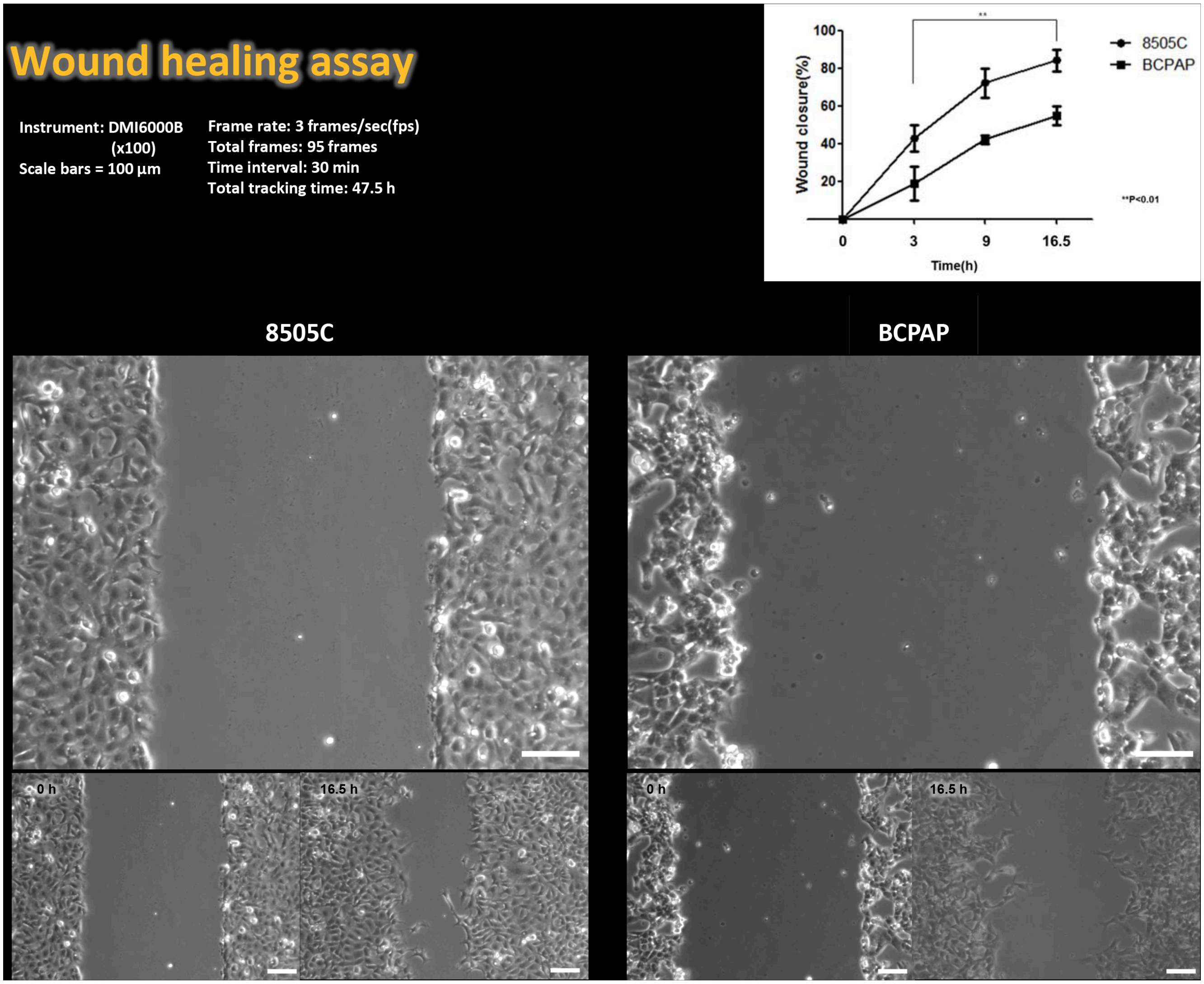

Supplementary Video S1: Live wound healing assay of 8505C and BCPAP after treatment with 1 µM PLX4032 for 9 h. $^{**}p < 0.01$.
